# High expression of STAT3 within the tumour‐associated stroma predicts poor outcome in breast cancer patients

**DOI:** 10.1002/cam4.6014

**Published:** 2023-05-18

**Authors:** Elizabeth Morrow, Kathryn Pennel, Phimmada Hatthakarnkul, Holly Leslie, Elizabeth Mallon, Ditte Andersen, Nigel Jamieson, Donald McMillan, Antonia Roseweir, Joanne Edwards

**Affiliations:** ^1^ School of Cancer Sciences, Wolfson Wohl Cancer Research Centre University of Glasgow Glasgow UK; ^2^ Biomedical Science Program, Faculty of Medicine Siriraj Hospital University of Mahidol Bangkok Thailand; ^3^ Department of Pathology Queen Elizabeth University Hospital Glasgow UK; ^4^ BioClavis Ltd, Queen Elizabeth University Hospital Glasgow UK; ^5^ Academic Unit of Surgery, Glasgow Royal Infirmary Glasgow UK; ^6^ School of Medicine, Wolfson Medical Building University of Glasgow Glasgow UK

**Keywords:** breast cancer, JAK/STAT signal transduction, prognostic markers, spatial profiling, STAT3, Stroma, tumour microenvironment

## Abstract

**Introduction:**

Triple‐negative breast cancer (TNBC) patients have the poorest clinical outcomes compared to other molecular subtypes of breast cancer. IL6/JAK/STAT3 signalling is upregulated in breast cancer; however, there is limited evidence for its role in TNBC. This study aimed to assess the expression of IL6/JAK/STAT3 in TNBC as a prognostic biomarker.

**Methods:**

Tissue microarrays consisting of breast cancer specimens from a retrospective cohort (*n* = 850) were stained for IL6R, JAK1, JAK2 and STAT3 via immunohistochemistry. Staining intensity was assessed by weighted histoscore and analysed for association with survival/clinical characteristics. In a subset of patients (*n* = 14) bulk transcriptional profiling was performed using TempO‐Seq. Nanostring GeoMx® digital spatial profiling was utilised to establish the differential spatial gene expression in high STAT3 tumours.

**Results:**

In TNBC patients, high expression of stromal STAT3 was associated with reduced cancer‐specific survival (HR = 2.202, 95% CI: 1.148–4.224, log rank *p* = 0.018). TNBC patients with high stromal STAT3 had reduced CD4^+^ T‐cell infiltrates within the tumour (*p* = 0.001) and higher tumour budding (*p* = 0.003). Gene set enrichment analysis (GSEA) of bulk RNA sequencing showed high stromal STAT3 tumours were characterised by enrichment of IFNγ, upregulation of KRAS signalling and inflammatory signalling Hallmark pathways. GeoMx™ spatial profiling showed high stromal STAT3 samples. Pan cytokeratin (panCK)‐negative regions were enriched for CD27 (*p* < 0.001), CD3 (*p* < 0.05) and CD8 (*p* < 0.001). In panCK‐positive regions, high stromal STAT3 regions had higher expression of VEGFA (*p* < 0.05).

**Conclusion:**

High expression of IL6/JAK/STAT3 proteins was associated with poor prognosis and characterised by distinct underlying biology in TNBC.

## Graphical abstract

This study has revealed a profound association between high protein expression of IL6/JAK/STAT3 signalling and worse clinical outcomes in a large retrospective cohort of breast cancer patients. Nanostring GeoMx® digital spatial profiling of triple negative breast cancers showed that high STAT3 cases were underpinned by upregulation of VEGFA in pancytokeratin positive regions and upregulation of CD27, CCL5, CD3 and CD8 in pan‐cytokeratin negative regions.

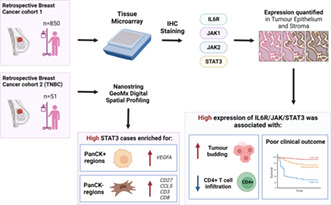

## 1 INTRODUCTION

Breast cancer is the most commonly diagnosed cancer and the second most common cause of cancer‐related mortality amongst females worldwide.^1^ Despite the recognition of molecular and histological subtypes, the development of novel prognostic and predictive biomarkers remains an important area of research due to the heterogeneous nature of tumours and lack of targeted treatment options for patients with triple‐negative breast cancer (TNBC). Tumours classified as triple negative lack expression of oestrogen (ER) and progesterone receptors and human epidermal growth factor 2 (HER2), and the mainstay of systemic treatment remains chemotherapy. TNBC cases make up approximately 15% of all breast cancers and have the poorest clinical outcomes.^1^ Research is focused on establishing the biological mechanisms responsible for driving tumour development and progression of TNBC. Identification of dysregulated cellular signalling pathways in TNBC will highlight new therapeutic targets for improving patient outcomes in this subtype of breast cancer.

The IL6/JAK/STAT3 pathway is an inflammatory signalling cascade involved in normal homeostatic processes, including immune cell differentiation, inflammation and tissue repair.^2^ In cancer cells signalling is constitutively active.^3^ Signal transduction can occur via the classical or *trans*‐signalling mechanisms, which both involve IL6 binding to its cognate receptor, IL6R, either membrane‐bound or in soluble form, respectively.^4^ This interaction causes phosphorylation of downstream proteins, mainly JAK1 and JAK2, which results in activation of STAT3 at tyrosine 705 in the cytoplasm. STAT3 then dimerises and translocates to the nucleus where it acts as a transcription factor. In pancreatic, colorectal and prostate cancer, high expression of IL6/JAK/STAT3 pathway proteins associates with poor clinical outcomes.^5^, ^6^ STAT3 is responsible for driving transcription of genes, which drive many of the hallmarks of cancer.^7^

In breast cancer, STAT3 activation has been associated with resistance to apoptosis, hypoxia, immune evasion and metastases in vitro.^8^, ^9^, ^10^ A role for STAT3 in facilitating invasion and metastases has also been identified using mouse models of breast cancer.^11^, ^12^ There is limited evidence in the literature to date which explores IL6/JAK/STAT3 as prognostic or predictive biomarkers in breast cancer. A small study of a retrospective cohort of 102 breast cancer patients identified an association between high expression of STAT3 within tumour cells with reduced overall survival.^13^ The aim of this study was to establish a prognostic role for IL6/JAK/STAT3 signalling pathway members in a larger retrospective cohort of breast cancer patients (*n* = 850) with a focus on TNBC. This study also aimed to describe differences in the underlying biology of tumours with high protein expression of IL6/JAK/STAT3 using TempO‐Seq transcriptional analysis in a subset of patients (*n* = 14) and histological scores of the tumour microenvironment (TME) in the full cohort.^14^

## 2 METHODS

### 2.1 Patient cohort characteristics

A previously constructed tissue microarray (TMA) consisting of a retrospective cohort of 850 breast cancer patients was utilised for the study (Cohort 1). Patients included underwent surgical resection with curative intent at Glasgow Royal Infirmary, Glasgow Western Infirmary or the Victoria Hospital Glasgow between 1995 and 2001 (Glasgow Safehaven number GSH/18/ON/008). Ethical approval was acquired from Multicentre Research Ethics Committee for Scotland and Local Research and Ethics Committees. Patients were excluded from the study if they died within 30 days of surgery or survival data were missing, and only ductal cases were included. This left 702 patients included in the study (Figure S1). At the time of last follow‐up 406 (57.8%) patients were alive, 161 (22.9%) patients had died of primary breast cancer and 135 (19.2%) had died of another cause. Mean survival time was 121 months. Invasive tumour grade was Grade I in 122 cases (17.4%), Grade II in 297 cases (42.3%) and Grade III in 283 cases (40.3%). There were 212 (30.2%) patients under 50 years old and 490 (69.8%) patients ≥50 years of age at presentation. Of these patients, 325 (43.2%) had Luminal A disease, 150 (22.7%) had Luminal B disease, 155 (23.5%) were classified as triple negative and 70 (10.6%) were HER‐2 enriched cases.

A second cohort (Cohort 2) consisting of TNBC patients was utilised for the spatial transcriptomic profiling performed in the study. This cohort consisted of 207 breast cancer patients classified as triple negative subtype (Glasgow Safehaven number GSH/21/ON/008). At the time of last follow‐up 128 patients were alive (62.1%), 69 (33.5%) had died of cancer and 2 (4.4%) had died of other causes in this cohort. The mean survival time was 51.1 months.

### 2.2 Immunohistochemistry

Immunohistochemical staining was performed manually. Prior to staining TMA sections were baked at 60°C for 20 min. Sections were dewaxed in Histo‐Clear (#NAT1334, National Diagnostics) and rehydrated through a series of graded alcohols. Antigen retrieval was performed by heating sections under pressure in citrate buffer pH 6 for JAK1 and JAK2 staining and in Tris‐EDTA pH 8 buffer for STAT3. Endogenous peroxidases were blocked in 3% H_2_O_2_ and non‐specific binding was blocked by incubating sections in 10% horse serum for 30 min at room temperature. Antibodies were added; JAK1 (1:200, Cell Signaling #3344), JAK2 (1:200, Cell Signaling #3230) or STAT3 (1:300, Cell Signaling #9139), respectively and incubated overnight at 4°C. Secondary antibody Envision (#K5007, Dako) was applied to sections for 30 min. After washing in TBS, 3,3′‐diaminobenzidine (DAB) (#SK‐4100, Vector) was added for 5 min. Sections were washed in water, counterstained and dehydrated through a series of graded alcohols. Sections were placed in Histo‐Clear (#NAT1334, National Diagnostics) and mounted using Omnimount histological mounting medium (#HS‐110, National diagnostics). Stained sections were scanned onto NDP viewer (Hamamatsu) using a Nanozoomer (Hamamatsu) for visualisation.

### 2.3 Scoring methods

For each patient, three cores of 0.6 μM diameter were included in the TMA to account for tumour heterogeneity. Protein expression levels were quantified by weighted histoscore (WHS) at 20× magnification by a single scorer (EM) blinded to clinicopathological data and outcomes. The proportion of cells with no staining (score 0), weak staining (score 1), moderate staining (score 2) and strong staining (score 3) was recorded. The WHS was calculated as (0x proportion score 0) + (1x proportion score 1) + (2x proportion score 2) + (3x proportion score 3). A separate score was recorded for tumour nuclear staining (STAT3), tumour cytoplasmic staining (JAK1/JAK2/STAT3) and stromal cell staining (JAK1/JAK2/STAT3). For validation purposes, 10% of cores were co‐scored independently by a second observer (AR) and the correlation coefficient was ≥0.7.

### 2.4 Mutational analyses

A publicly available dataset (MSK Cancer Cell A Breast cancer cohort, *n* = 1918) was analysed for the presence of JAK1 and JAK2 mutation online at cbioportal.org. Mutation status was assessed for association with clinical outcome and characteristics.

### 2.5 TempO‐Seq® bulk RNA sequencing

Full transcriptomic profiling data were available for 14 patients with TNBC from the retrospective cohort. This was performed using formalin‐fixed paraffin‐embedded sections utilising the TempOSeq platform (BioSpyder). Profiling was performed as previously described.^14^ Normalisation of read counts was performed using DESeq2 in R Studio v 3.1 (RStudio). GSEA was performed using publicly available software (https://www.gsea‐msigdb.org/gsea/index.jsp) using the Hallmark database. A heatmap of the top 50 upregulated and downregulated genes was constructed using GSEA application.

### 2.6 GeoMx™ digital spatial profiling

The GeoMx digital spatial profiling (DSP) platform provided a robust detection of high‐plex RNA expression from user‐defined compartments within FFPE tissues. Tissue arrays from Cohort 2 (TNBC cohort) (*n* = 51) were cut at 5 μm and baked for 30 min at 60°C. This TMA consisted of three cores of 0.6 μM diameter per patient. Epitope retrieval was performed in Leica BOND autostainer (ER2, pH 9, 100°C) for 10 min and protein digestion using proteinase K (0.1 μg/mL) for 15 min. In situ hybridisation of RNA‐directed DNA oligo probes (Immune Pathways Panel, Nanostring) for 84 gene targets was performed according to the manufacturer's protocol. HybriSlip™ covers were applied prior to overnight incubation at 37°C for at least 16 h (Thermofisher). Slides were then washed twice with a 1:1 ratio of 100% deionised formamide (Ambion Inc.) and 4X SSC (Sigma Aldrich) at 37°C for 25 min. Immunofluorescent staining was performed using primary conjugated antibodies (Pan‐Cytokerratin (panCK), CD45) and nucleic acid dye (SYTO 13) and then stored at 4°C in SSC before being loaded on the GeoMx DSP instrument for visualisation, region of interest (ROI) selection and collection. ROIs were selected based on successful 3‐plex immunofluorescence staining of SYTO 13, panCK and CD45. Circular ROIs were drawn incorporating TMA cores with areas of interest selected according to panCK staining; with the panCK‐positive mask and panCK‐negative mask identifying tumour and stromal areas, respectively (Figure S1). The barcodes, conjugated with the selected antibody, were then collected upon cleavage with a UV light using the GeoMX instrument before processing using Nanostrings MAX/FLEX nCounter system.

The nCounter readout of GeoMx DSP‐collected probes was performed as per the manufacturer's protocol (NanoString Technologies). Data acquisition was performed by using the Nanostring's Digital Analyser (FOV, 555). Output files (.dcc) were then uploaded back on to the GeoMx built‐in analysis suite for preliminary analysis and data quality check (QC) by two observers (HL, PH). Counts were normalised with negative probes using the geometric mean. Data were extracted and the cut‐off threshold for high and low expression was determined using R Studio version 4.0.5 (Rstudio) for both panCK+ and panCK− compartments.

### 2.7 Statistical analyses

The cut‐offs for high and low expression were generated in R Studio version 4.0.5 (RStudio) using maxstat, survminer and tidyverse packages. This generated the optimal cut‐off point based on cancer‐specific survival (CSS) data using log rank statistics. Kaplan–Meier survival analysis was performed in SPSS version 27 (IBM) to determine association with clinical outcomes. Cox regression and life tables were generated in SPSS. Non‐parametric Kruskal–Wallis testing was used to assess association between discrete protein expression groups and continuous variables associated with the characterisation of the TME and bar charts were plotted using GraphPad Prism version 8 (GraphPad Software Inc.). Analysis of the publicly available MSK Cancer Cell breast cohort (*n* = 1918) mutational dataset was performed using cBioPortal (https://www.cbioportal.org/). For GeoMx™ data, volcano plots and box plots were constructed in R studio using DESeq2 and ggplot packages and GraphPad Prism version 8 (GraphPad Software Inc.). Significance was set to *p* < 0.05.

## 3 RESULTS

### 3.1 High expression of IL6R within the tumour cell cytoplasm and surrounding stroma was associated with poor outcome in breast cancer patients

**FIGURE 1:**
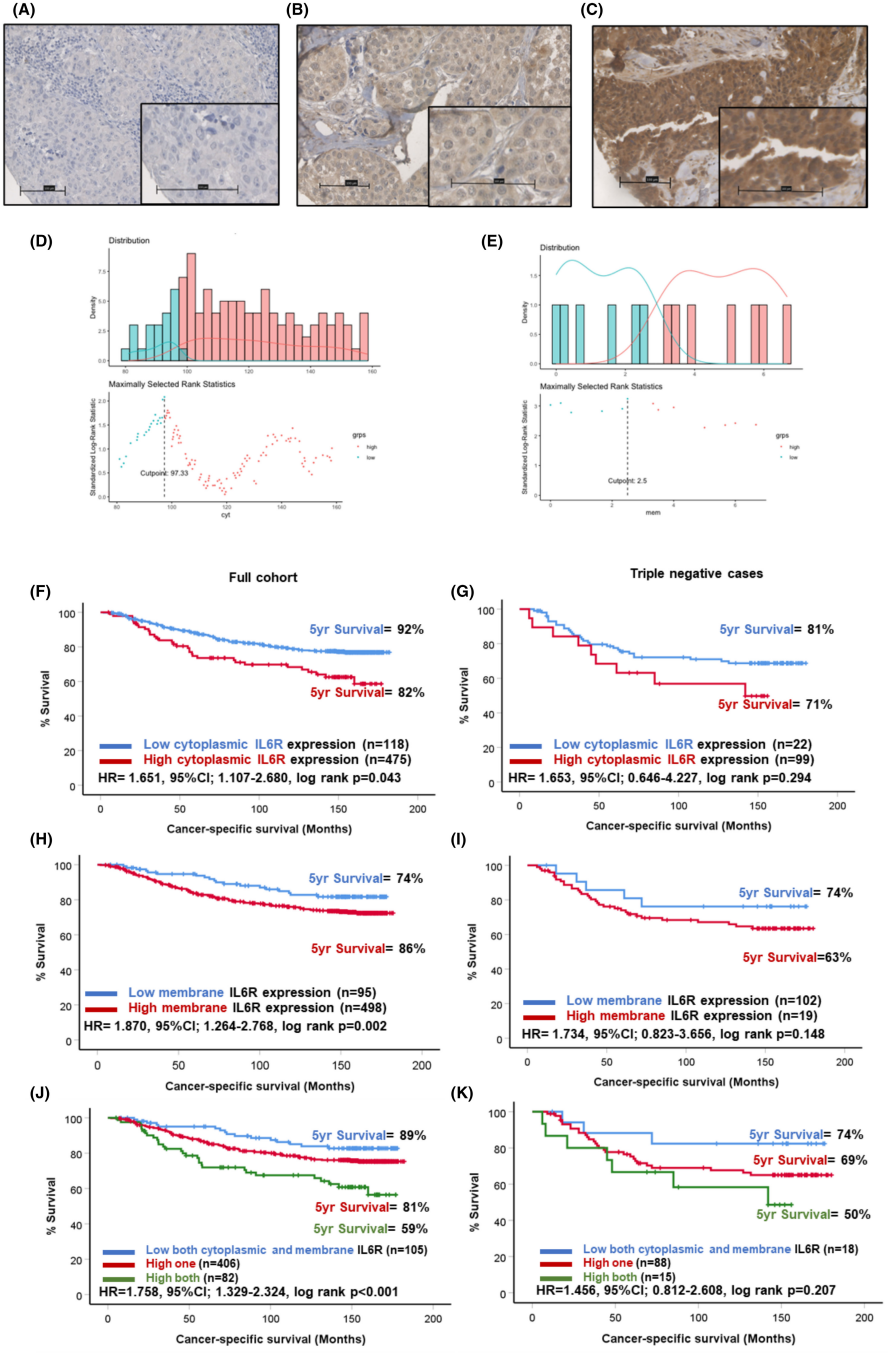
Expression of IL6R in breast cancer. Representative images showing weak (A), moderate (B) and strong (C) staining of IL6R in tumour cells. Histograms showing the distribution of cytoplasmic (C) and membranous (D) IL6R scores and cut‐offs for high and low expression. Kaplan–Meier curves showing the association between cytoplasmic IL6R expression and cancer‐specific survival across all breast cancer subtypes (F) and in triple negative cases (G). Kaplan–Meier curves showing the association between membranous IL6R expression and cancer‐specific survival across all breast cancer subtypes (H) and in triple negative cases (I). Kaplan–Meier curve showing the association between combined cytoplasmic and membranous IL6R expression and cancer‐specific survival across all breast cancer subtypes (J) and in triple negative cases (K).

Positive expression of IL6R staining was detected in the tumour cell cytoplasm and membrane (Figure 1A–C). Scores for tumour cytoplasmic IL6R were available for 591 patients from Cohort 1 (Figure S1). Scores ranged from 0 to 300 with a mean score of 112.91. The expression values for tumour cytoplasmic IL6R were not significantly different across the molecular subtypes of breast cancer (*p* = 0.230) (Figure S2A). An optimal cut‐off point of 97.33 was determined by log rank statistics resulting in 113 (20%) patients classified as low and 465 (80%) classified as high for cytoplasmic IL6R (Figure 1D). Scores for tumour membrane IL6R were available for 578 patients (Figure S1) and ranged from 0 to 150 with a mean of 3.84. The optimal cut‐off point for high and low expression was 2.5 and this resulted in 486 (84%) patients classified as low and 92 (16%) patients classified as high for membranous IL6R (Figure 1E). Kaplan–Meier survival analysis showed a significant association between high cytoplasmic IL6R expression and reduced CSS in the full cohort (HR = 1.651, 95% CI: 1.107–2.680, log rank *p* = 0.043) (Figure 1F). There were 92% of patients with low cytoplasmic IL6R expression alive at 5 years post‐surgery compared to 82% of patients classified as high for cytoplasmic IL6R (Figure 1F). When triple negative patients were analysed independently, a similar trend was observed; however, statistical significance was lost (Figure 1G).

The expression values for tumour membrane IL6R were not significantly different across the molecular subtypes of breast cancer (*p* = 0.714) (Figure S2B). In the full cohort Kaplan–Meier survival analysis showed high tumour cell membranous expression of IL6R was associated with reduced CSS (HR = 1.870, 95% CI: 1.264–2.768, log rank *p* = 0.002) (Figure 1H). Only 74% of patients classified as high for tumour cell membrane IL6R were alive 5 years post‐surgery compared to 86% of patients classified as low for membrane IL6R (Figure 1H). A similar trend was observed when the triple negative cases were analysed separately; however, statistical significance was lost (Figure 1I). When scores for tumour cytoplasmic and membrane IL6R were combined to form three groups of low for both, high for one marker and high for both markers, a significant association with reduced CSS in the high both group was observed (HR = 1.758, 95% CI: 1.329–2.324, log rank *p* < 0.001) (Figure 1J). This trend continued when the tripe negative cases were analysed; however, statistical significance was lost (Figure 1K).

### 3.2 High expression of JAK1 within the tumour cytoplasm associated with poor prognosis in triple‐negative breast cancer

**FIGURE 2:**
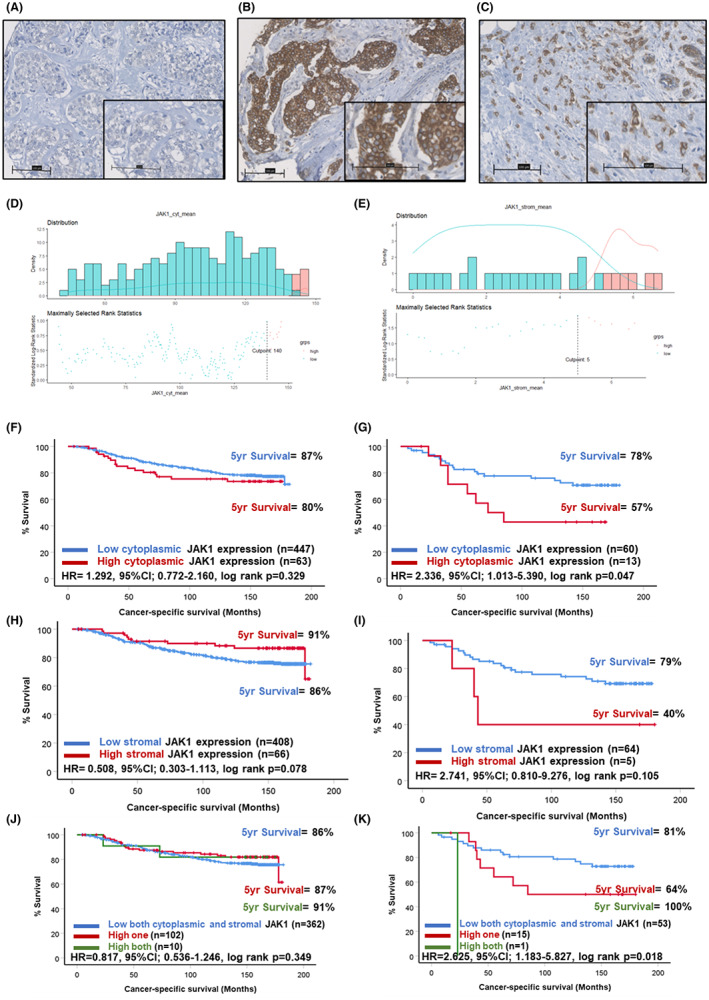
Expression of JAK1 in breast cancer. Representative images showing negative (A) and strong tumour cytoplasmic (B) staining of JAK1. Representative image of positive staining for JAK1 in the tumour‐associated stroma (C). Histograms showing the distribution of tumour cytoplasmic (C) and stromal (D) JAK1 scores and cut‐offs for high and low expression. Kaplan–Meier curves showing the association between tumour cytoplasmic JAK1 expression and cancer‐specific survival across all breast cancer subtypes (F) and in triple negative cases (G). Kaplan–Meier curves showing the association between stromal JAK1 expression and cancer‐specific survival across all breast cancer subtypes (H) and in triple negative cases (I). Kaplan–Meier curve showing the association between combined tumour cytoplasmic and stromal JAK1 expression and cancer‐specific survival across all breast cancer subtypes (J) and in triple negative cases (K).

JAK1 staining was identified in the tumour cell cytoplasm of a subgroup of patients, with representative images of negative and positive staining shown in Figure 2A,B. Staining was also detected in the tumour‐associated stroma in a subset of patients (Figure 2C). When staining was quantified in the tumour cell cytoplasm scores ranged from 0 to 230 with a median score of 101.67. Scores for cytoplasmic JAK1 were available for 508 patients (Figure S1). The scores for JAK1 in tumour cytoplasm (*p* = 0.022) and stromal expression (*p* = 0.045) were significantly different when compared across molecular subtypes of breast cancer (Figure S2C,D). An optimal cut‐off point of 140 was calculated for cytoplasmic JAK1 as shown in the histogram (Figure 2D). This resulted in 431 (88%) patients classified as low JAK1 and 61 (12%) as high for JAK1 expression. Scores for stromal JAK1 were available for 471 patients (Figure S1) and ranged from 0 to 130 with a median score of 1.67. An optimal cut‐off point of 5 was devised for stromal JAK1 (Figure 2E). This resulted in 391 (86%) patients classified as low and 66 (14%) as high for stromal JAK1. Kaplan–Meier survival analysis demonstrated no association between cytoplasmic expression of JAK1 and CSS in the full cohort (Figure 2F); however, there was a significant association between high JAK1 expression and reduced CSS in patients with triple negative disease (Figure 2G). In triple negative patients, 78% of the low cytoplasmic JAK1 group were alive at 5 years post‐surgery compared to 57% of patients classified as high for cytoplasmic JAK1 (HR = 2.336, 95% CI: 1.013–5.390, log rank *p* = 0.047). There was no significant association between stromal expression of JAK1 in the full cohort (Figure 2H), or in triple negative cases (Figure 2I). When stromal and tumour cytoplasmic JAK1 were combined to form a score of both low (0), one high^1^ or both high,^5^ there was no association between this score and CSS in the full cohort (Figure 2J). In triple negative cases, patients low for both markers had significantly improved outcomes (HR 2.625, 95% CI: 1.183–5.827, log rank *p* = 0.018) (Figure 2K).

### 3.3 High expression of JAK2 within the tumour cytoplasm associated with poor prognosis in triple‐negative breast cancer

**FIGURE 3:**
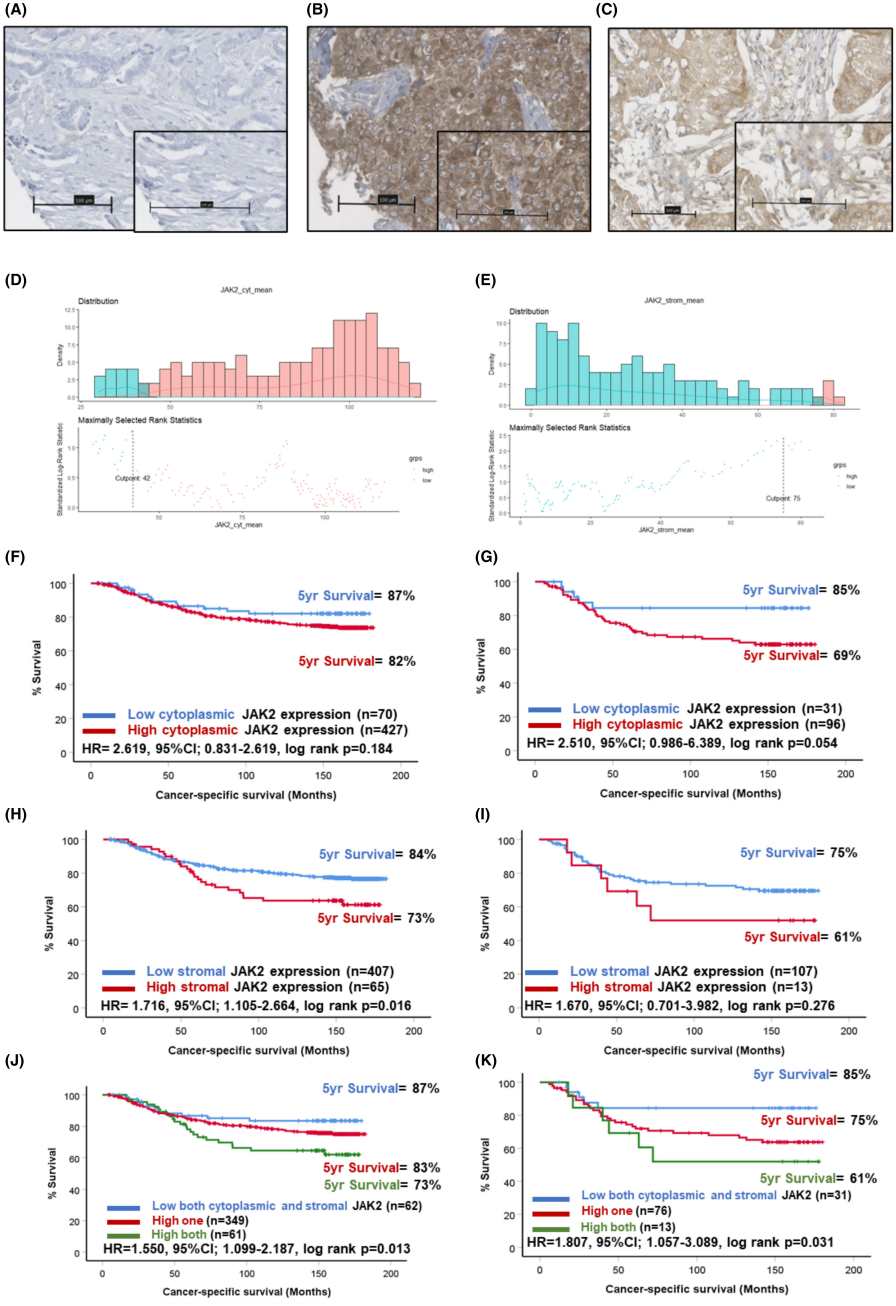
Expression of JAK2 in breast cancer. Representative images showing negative (A) and strong tumour cytoplasmic (B) staining of JAK2. Representative image of positive staining for JAK2 in the tumour‐associated stroma (C). Histograms showing the distribution of tumour cytoplasmic (C) and stromal (D) JAK2 scores and cut‐offs for high and low expression. Kaplan–Meier curves showing the association between tumour cytoplasmic JAK2 expression and cancer‐specific survival across all breast cancer subtypes (F) and in triple negative cases (G). Kaplan–Meier curves showing the association between stromal JAK2 expression and cancer‐specific survival across all breast cancer subtypes (H) and in triple negative cases (I). Kaplan–Meier curve showing the association between combined tumour cytoplasmic and stromal JAK2 expression and cancer‐specific survival across all breast cancer subtypes (J) and in triple negative cases (K).

Similarly, JAK2 staining was present within the tumour cell cytoplasm and stromal areas of some patients as shown in representative images (Figure 3A–C). Scores for tumoural cytoplasmic JAK2 were available for 495 patients (Figure S1) and ranged from 0 to 195 with a median of 99.0. The scores for both cytoplasmic and stromal JAK2 differed significantly across molecular subtypes of breast cancer (*p* < 0.001) (Figure S2E,F). The optimal cut‐off point for high and low expression groups determined was 42.0, which resulted in 81 (15%) patients classified as low for JAK2 expression and 479 (85%) classified into the high group (Figure 3D). Scores for stromal JAK2 were available for 471 patients (Figure S1) and ranged from 0 to 193 with a median of 20.5. An optimal cut‐off point of 75 was determined, which resulted in 457 (87%) classified as low and 70 (13%) as high for stromal JAK2 (Figure 3E). Kaplan–Meier survival analysis showed no significant association between cytoplasmic JAK2 expression and outcome in the full cohort (Figure 3F). There was a trend towards high cytoplasmic JAK2 expression predicting poorer prognosis when triple negative patients were assessed (HR = 2.510, 95% CI: 0.986–6.389, log rank *p* = 0.054) (Figure 3G). Kaplan–Meier survival analysis revealed a significant association between high stromal JAK2 expression and reduced CSS in the full cohort (HR = 1.716, 95% CI: 1.105–2.664, log rank *p* = 0.016). There were 84% of the low stromal JAK2 group alive at 5 years post‐surgery compared to 73% of the high expression group (Figure 3H). A similar trend was observed for triple negative cases; however, statistical significance was lost (Figure 3I). When scores for tumour cytoplasm and stromal JAK2 were combined, patients high for the marker in both locations had significantly reduced prognosis (HR = 1.550, 95% CI: 1.099–2.187, log rank *p* = 0.013) (Figure 3J). This was also seen in the triple negative cases specifically (HR = 1.807, 95% CI: 1.057–3.089, log rank *p* = 0.031) (Figure 3K). In these patients, there were 85% of the low both markers group alive at 5 years post‐surgery compared to 75% in patients high for one marker and a drop to 61% in patients high for both tumoural cytoplasmic and stromal JAK2.

### 3.4 High expression of JAK1 and JAK2 within the tumour cytoplasm is associated with poor outcome

**FIGURE 4:**
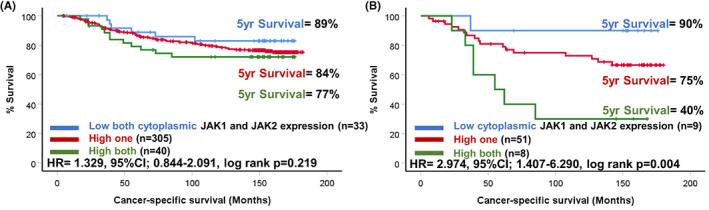
Expression of JAK1 and JAK2 in breast cancer. Kaplan–Meier curves showing the association between combined tumour cytoplasmic JAK1 and JAK2 scores and cancer‐specific survival across all breast cancer molecular subtypes (A) and in triple negative cases (B).

When scores for cytoplasmic JAK1 and JAK2 were combined there were 39 (9.3%) patients who were low for both markers, 338 (80.3%) high for one and 44 (10.5%) high for both markers. There was no association between the combined cytoplasmic JAK1/JAK2 score and CSS in the full cohort (4A). However, in triple negative cases there was a profound drop in survival amongst patients classified as high for both JAK1 and JAK2 compared to the other two groups (HR = 2.974, 95% CI: 1.407–6.290, log rank *p* = 0.004) (Figure 4B). In these triple negative cases there were 90% of patients classified as low for both markers alive at 5 years post‐surgery compared to 75% classified as high for one marker and only 40% of patients classified as high for both were alive.

### 3.5 High expression of STAT3 within the tumour‐associated stroma associated with reduced outcome in breast cancer

**FIGURE 5:**
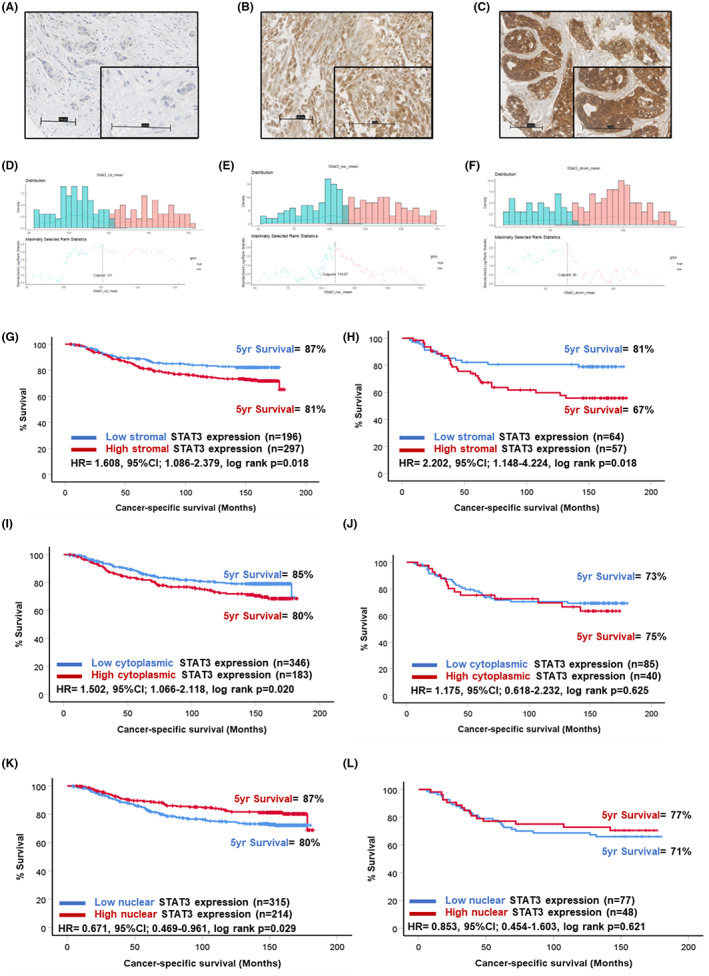
Expression of STAT3 in breast cancer. Representative images showing negative (A) and stromal (B) and tumoural (C) staining of STAT3. Histograms showing the distribution of tumour cytoplasmic (D), tumour nuclear (E) and stromal (F) STAT3 scores and cut‐offs for high and low expression. Kaplan–Meier curves showing the association between stromal STAT3 and cancer‐specific survival across all breast cancer subtypes (G) and in triple negative cases (H). Kaplan–Meier curves showing the association between cytoplasmic STAT3 expression and cancer‐specific survival across all breast cancer subtypes (I) and in triple negative cases (J). Kaplan–Meier curve showing the association between nuclear STAT3 expression and cancer‐specific survival across all breast cancer subtypes (K) and in triple negative cases (L).

Next, downstream signalling pathway member STAT3, known to be activated by JAK1 and JAK2, was assessed in the full cohort. STAT3 was detected in the tumour cell cytoplasm, nuclei and also in the tumour‐associated stroma. Representative images of staining patterns observed are shown in Figure 5A–C. Scores for stromal STAT3 were available for 492 patients (Figure S1) and ranged from 0 to 205 with a median score of 82.5. The scores for stromal (*p* = 0.001) and nuclear STAT3 (*p* = 0.001) varied significantly between molecular subtypes but not cytoplasmic expression (*p* = 0.068) of STAT3 (Figure S2G–I). The optimal cut‐off point determined was 66.0, which resulted in 216 (39%) classified as low and 339 (61%) as high for stromal STAT3 (Figure 5D). Scores for tumoural cytoplasmic STAT3 were available for 527 (Figure S1) patients and ranged from 0 to 209.33 with a median score of 114.3. The optimal cut‐off point determined was 121.0, which resulted in 401 (66%) of patients classified as low and 200 (24%) as high for cytoplasmic STAT3 (Figure 5E). Scores for nuclear STAT3 were available for 527 patients (Figure S1) and ranged from 0 to 260 with a median of 110.04. The optimal cut‐off point determined was 110.67, which resulted in 352 (59%) patients classified as low and 249 (41%) patients high for nuclear STAT3 expression (Figure 5F).

In the full cohort, Kaplan–Meier survival analysis showed high stromal STAT3 expression was associated with reduced CSS (HR = 1.608, 95% CI: 1.086–2.379, log rank *p* = 0.018) (Figure 5G). At 5 years post‐surgery, 87% of patients classified as low for stromal STAT3 were alive compared to 81% in the high stromal STAT3 group. This relationship was potentiated in triple negative cases (HR = 2.202, 95% CI: 1.148–4.224, log rank *p* = 0.018) (Figure 5H). There were 81% of triple negative patients classified as low alive 5 years post‐surgery compared to 67% of the high stromal STAT3 group. Kaplan–Meier survival analysis revealed high cytoplasmic expression of STAT3 within the tumour epithelium was associated with reduced outcome in the full cohort (HR = 1.502, 95% CI: 1.066–2.118, log rank *p* = 0.020) (Figure 5I). However, this relationship was not observed when the cohort was stratified into triple negative patients (Figure 4J). Converse to our hypotheses, Kaplan–Meier survival analysis showed low tumour nuclear (surrogate marker of activation) expression of STAT3 was significantly associated with reduced outcome in the full cohort (HR = 0.671, 95% CI: 0.469–0.961, log rank *p* = 0.029) (Figure 5K). This relationship was lost when triple negative cases were extracted and analysed separately (Figure 5L).

### 3.6 Distinct immune, histological and transcriptional profiles underlie high stromal STAT3 phenotypes in triple negative cases

**FIGURE 6:**
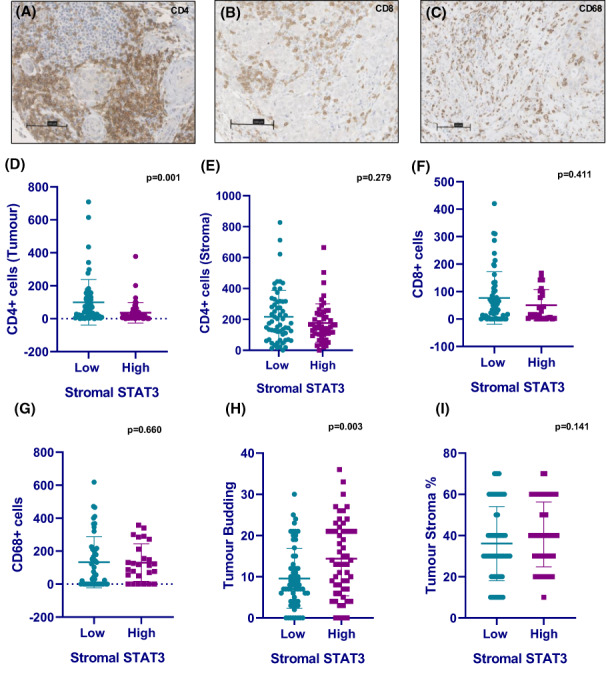
Immune profiles of high STAT3 in triple‐negative breast cancer cases. Representative images showing positive staining for CD4^+^ T cells (A), CD8^+^ T cells (B) and CD68^+^ macrophages (C) in TNBC tissue. Bar charts showing tumoural CD4^+^ stromal CD4^+^/CD8^+^/CD68^+^ cell counts in low and high stromal STAT3 TNBC cases (D–G). Bar charts showing tumour bud counts and tumour stroma percentage relative to stromal STAT3 status in TNBC (H, I).

**FIGURE 7:**
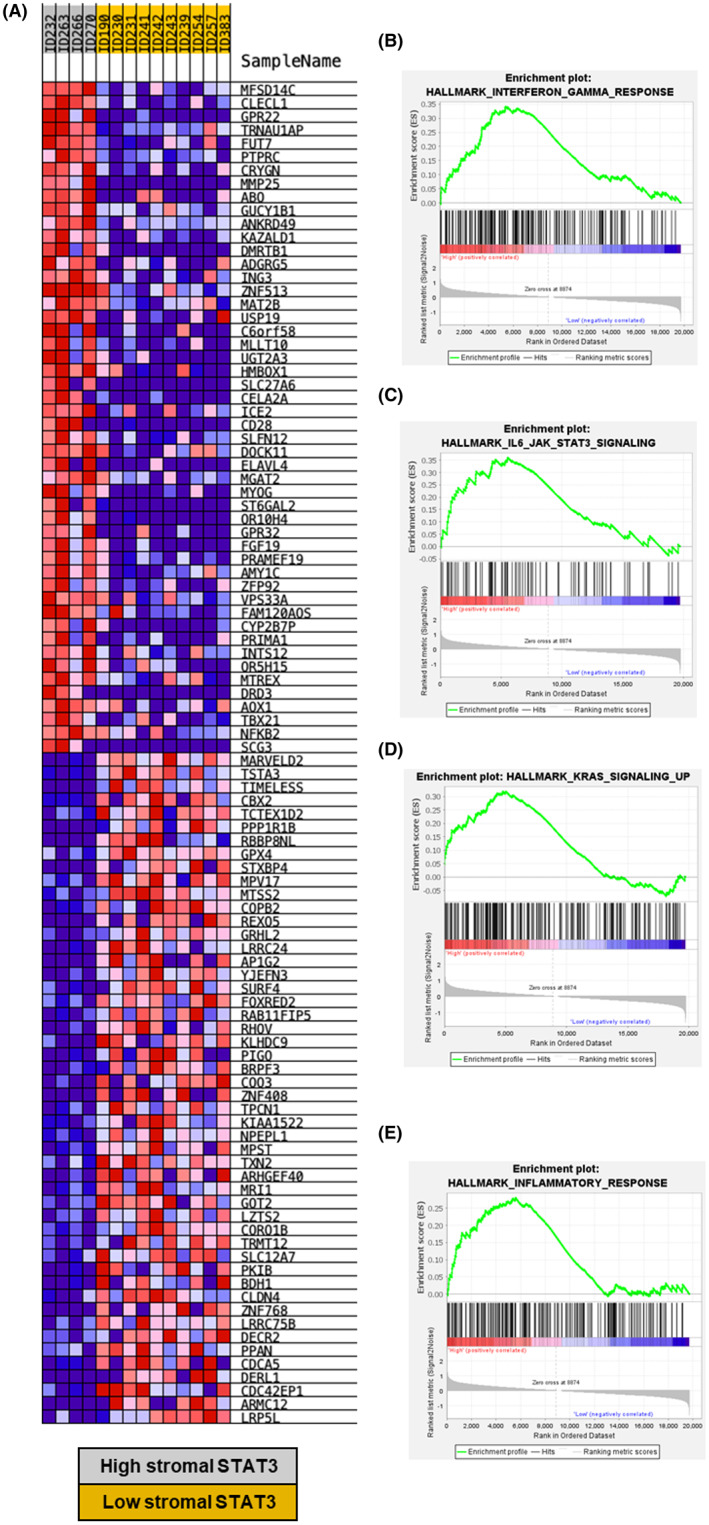
Gene expression analysis of high/low stromal STAT3 TNBC patients. Heatmap showing differential expression analysis between high stromal STAT3 (grey) and low stromal STAT3 (yellow) patients (A). Gene set enrichment analysis plots of high versus low stromal STAT3 cases showing enrichment of Hallmark interferon gamma signalling (B), IL6/JAK/STAT3 signalling (C), Hallmark KRAS signalling up (D) and Hallmark inflammatory signalling (E) in triple negative cases.

When the immune landscape was assessed by chromogenic IHC, positive staining was detected for CD4^+^ T cells, CD8^+^ T cells and CD68^+^ macrophages as shown in representative images (Figure 6A–C). Kruskal–Wallis non‐parametric tests showed patients with high stromal expression of STAT3 had reduced CD4^+^ T‐cell counts within the tumour (*p* = 0.001) but not the stroma (*p* = 0.279) in triple negative cases (Figure 6D,E). There was no association between CD8^+^ or CD68^+^ cell infiltrates and stromal STAT3 status (Figure 6F,G). However, there was an increased presence of tumour buds and higher tumour stroma percentage (TSP) detected in triple negative patients with high stromal STAT3 (Figure 6H,I). When the transcriptional profile of a subset of the cohort was assessed, data were available for 14 patients with triple negative disease. Of these patients, 4 were classified as high for stromal STAT3 and 10 low for stromal STAT3. A clear pattern of differences in the top 50 upregulated and downregulated genes amongst high versus low stromal STAT3 groups was observed upon heatmap construction (Figure 7A). When gene set enrichment analysis was performed using the Hallmark database, there was enrichment of Hallmark gene sets including interferon gamma response (ES = 0.34, FDR *q* = 0.06), IL6/JAK/STAT3 (ES = 0.36, FDR *q* = 0.012), upregulated KRAS signalling (ES = 0.32, FDR *q* = 0.0.01) and inflammatory response (ES = 0.28, FDR *q* = 0.046) (Figure 7B–E).

The Pearson correlation between stromal STAT3 and JAK2 scores in TNBC cases was assessed which yielded a moderate association (rho = 0.477, *p* < 0.001) as shown in a scatter plot (Figure S3A). When stromal JAK2 groups were analysed for association with immune infiltrates, there was significantly reduced CD4^+^ T cells in the tumour (*p* < 0.001) and no association with CD4^+^ cells in the stroma (*p* = 0.308) (Figure S3B,C).

### 3.7 High expression of STAT3 in the stroma is accompanied by spatially distinct differences in gene expression

**FIGURE 8:**
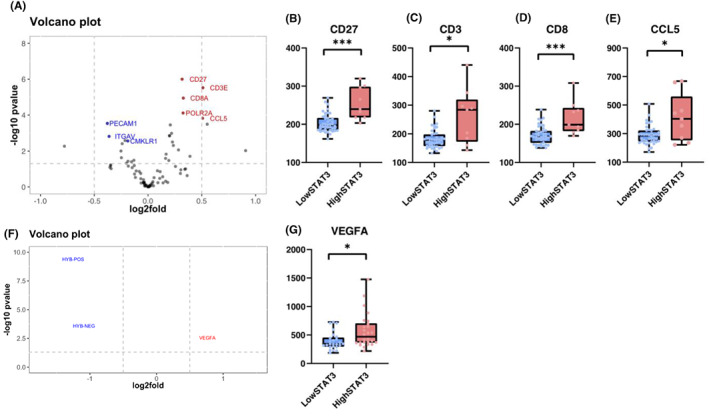
Digital spatial profiling of high/low stromal STAT3 TNBC patients. Volcano plot showing differential gene expression in high/low stromal STAT3 cases from Nanostrong GeoMx profiling of panCK‐negative regions of TNBC patients (*n* = 14) (A). Box plots showing expression of genes significantly enriched in high stromal STAT3 cases including CD27 (*p* = 0.0001), CD3 (*p* = 0.0141), CD8 (*p* = 0.0005) and CCL5 (*p* = 0.0397) (B–E). Volcano plot showing differentially expressed genes between high/low stromal STAT3 TNBC cases in panCK‐positive regions (F). Box plot showing the expression of VEGFA relative to stromal STAT3 status (*p* = 0.0110) (G). *p ≤ 0.05, **p ≤ 0.005, ***p ≤ 0.0005.

GeoMx™ profiling in panCK‐negative tissue showed differential gene expression between patients with high and low stromal STAT3 as represented in a volcano plot (Figure 8A). Gene expression of CD27 (*p* = 0.0001), CD3 (*p* = 0.0141), CD8 (*p* = 0.0005) and CCL5 (*p* = 0.0397) was higher in the high stromal STAT3 cases as represented in box plots (Figure 8B–E). In the panCK‐positive tissue there were fewer genes differentially expressed between high and low stromal STAT3 cases than in panCK‐negative tissue (Figure 8F). Expression of VEGFA (*p* = 0.0110) was higher in high stromal STAT3 tumours (Figure 8G).

### 3.8 The poor prognostic effect of JAK/STAT3 signalling is not mutationally driven in breast cancer

When a large publicly available cohort of patients (*n* = 1819) were assessed for the presence of mutations in the JAK1 and JAK2 genes, alterations were only detected in 1.8% of cases. The hotspot locations for mutations are represented by Lollipop plots shown in Figure S4A. The presence of an alteration in either or both genes was associated with higher tumour grade (*p* = 0.002) (Figure S4B). However, no association was identified between mutation presence and survival outcome upon Kaplan–Meier analysis (Figure S4C).

## 4 DISCUSSION

This retrospective study has demonstrated a prognostic role for JAK/STAT3 signalling in breast cancer with potentiation in TNBC. In particular, high STAT3 expression within the tumour‐associated stroma was associated with significantly reduced CSS (HR = 2.202, 95% CI: 1.148–4.224, log rank *p* = 0.018). The 5‐year survival of TNBC patients with high stromal STAT3 in this cohort was only 67% compared to 81% in patients with low stromal STAT3.

Previous studies have shown expression of STAT3 and activated STAT3 (pSTAT3) within tumour cells to be associated with outcome in colorectal, pancreatic and prostate cancers; however, there is limited evidence in the literature exploring STAT3 expression within the stroma.^5^, ^6^, ^15^ The presence of a high tumour stromal volume is associated with poor outcome in breast cancer as a whole and in TNBC cases.^16^, ^17^ This highlights the potential importance of investigating factors within the tumour‐stroma, which may be responsible for driving tumour development and progression.

Recent evidence from in vitro and in vivo models of breast cancers have highlighted STAT3 as a prominent mediator involved in the pro‐tumorigenic function of cancer‐associated fibroblasts (CAFs).^18^ In pancreatic cancer models, STAT3 activation in CAFs promotes an immunosuppressive phenotype.^19^ In the present study, high stromal STAT3 was associated with reduced CD4^+^ T‐cell infiltrates and increased TSP. Further work is required to characterise this in vitro/in vivo to determine whether high STAT3 within stromal cells is dampening immune responses within the TME.^19^

In addition to modulation of histological features of the TME, upregulation of KRAS signalling was enriched at the transcriptomic level in the tumours with high stromal STAT3.^20^ This corroborates data from PDAC models, which have shown that inhibition of downstream KRAS signalling member MEK combined with STAT3 resulted in stromal remodelling and induced anti‐tumour immune responses.^21^ Spatially resolved transcriptomic analysis showed that within panCK‐negative tissue there was enrichment of genes including CD27 (*p* = 0.0001), CD3 (*p* = 0.0141), CD8 (*p* = 0.005) and CCL5 (*p* = 0.0397) in high stromal STAT3 cases. CD27 is an upstream member of the NF‐κB pathway, which may indicate crosstalk with JAK/STAT3 signalling, which has previously been identified in several solid tumour types.^22^ Within panCK‐positive tissues high STAT3 within the stroma was associated with increased expression of VEGFA (*p* = 0.0110), which has been shown to be independently prognostic in TNBC and associated with more aggressive tumour phenotypes.^23^

In addition to stromal STAT3, high JAK1 and JAK2 expression within the tumour cell cytoplasm was associated with reduced CSS in TNBC cases. The 5‐year survival of TNBC patients classified as high for both cytoplasmic JAK1 and JAK2 was only 40%, compared to 90% in patients low for both markers (HR = 2.974, 95% CI: 1.407–6.290, log rank *p* = 0.004). Studies in the literature have focused on mRNA expression of JAK1 and JAK2 and have concluded high expression predicts better outcome in breast cancer.^24^ However, our findings at the protein level of JAK1/2 as poor prognostic markers support findings from other tumour types, including ovarian, non‐small cell lung cancer and pancreatic cancer.^25^, ^26^ IL6R expression within tumour cell membranes and cytoplasm was associated with reduced CSS in the full cohort but statistical significance was lost when TNBC were extracted and analysed. This finding suggests other upstream activators of STAT3 should be investigated in TNBC such as epidermal growth factor, interleukin‐10, interleukin‐11 and interleukin‐23.^4^

In the literature there are conflicting data regarding the prognostic nature of STAT3 within tumour nuclei. Our finding that low nuclear STAT3 expression predicted poorer outcomes was contrary to our hypothesis; however, a number of previous studies corroborate these findings. In a study of 346 node‐negative cases, high nuclear STAT3 and pSTAT3^tyr705^ associated with significantly increased survival.^27^ Further work is required to understand this phenomenon as nuclear localisation of STAT3 is a surrogate marker for pathway activation. Assessment of STAT3 phosphorylation sites (Tyrosine 705 and Serine 727) by IHC should be investigated in this cohort as a true measure of pathway activation. Dual immunofluorescent staining of STAT3 and pSTAT3 could be performed to assess if co‐localisation results in an association with reduced survival outcomes.

Given the association with poor outcome, inhibiting JAK/STAT3 signalling represents a promising therapeutic strategy for investigation in breast cancer. A number of studies have investigated this in vitro. Pathway inhibition using the FDA‐approved drug ruxolitinib that targets JAK1 and JAK2 inhibited growth and vascular endothelial growth factor expression in MCF‐7 cell lines.^28^ In four TNBC cell lines, inhibiting STAT3 using LLY17 reduced cell migration, growth and increased susceptibility to apoptosis in vitro.^29^ In ER‐positive breast cancer, there are studies looking at JAK inhibitors such as ruxolitinib to treat systemic inflammation.^30^ Further research is needed to validate these findings with an approach using more recapitulative models of patient disease such as patient‐derived organoids and co‐cultures with CAFs.

This study has demonstrated a significant association between high expression of STAT3 within the tumour‐associated stroma and reduced CSS in patients with TNBC. The high stromal STAT3 phenotype was characterised by a distinct stromal dense immunologically cold TME and differential gene expression profile. Future studies should look to investigate the therapeutic potential of inhibiting STAT3 in models of TNBC.

## AUTHOR CONTRIBUTIONS

**Elizabeth Morrow:** Conceptualization (equal); data curation (equal); formal analysis (equal); investigation (equal); methodology (equal); project administration (equal); writing – original draft (equal); writing – review and editing (equal). **Kathryn Pennel:** Formal analysis (equal); writing – original draft (equal); writing – review and editing (equal). **Phimmada Hatthakarnkul:** Formal analysis (equal); writing – original draft (equal); writing – review and editing (equal). **Holly Leslie:** Data curation (equal); methodology (equal); project administration (equal). **Elizabeth Mallon:** Investigation (equal); methodology (equal); supervision (equal); validation (equal). **Ditte Andersen:** Data curation (equal); resources (equal); software (equal); supervision (equal); writing – review and editing (equal). **Nigel Jamieson:** Data curation (equal); methodology (equal); project administration (equal); resources (equal); supervision (equal); writing – review and editing (equal). **Donald C McMillan:** Conceptualization (equal); funding acquisition (equal); supervision (equal); writing – review and editing (equal). **Antonia K. Roseweir:** Conceptualization (equal); data curation (equal); formal analysis (equal); investigation (equal); supervision (equal); validation (equal). **Joanne Edwards:** Conceptualization (equal); formal analysis (equal); funding acquisition (equal); methodology (equal); project administration (equal); supervision (equal); validation (equal); writing – review and editing (equal).

## FUNDING INFORMATION

This work was supported by funding from a Medical Research Council Transitional Fellowship for Kathryn Pennel (MR/R502327/1).

## Supporting information


Figure S1:

Figure S2:

Figure S3:

Figure S4:

## Data Availability

Glasgow Safehaven numbers GSH/18/ON/008GSH/21/ON/008.
