# Supplementary figures and images for: High expression of STAT3 within the tumour‐associated stroma predicts poor outcome in breast cancer patients
Source: Cancer Med. 2023 May 18;12(12):13225–40. doi: 10.1002/cam4.6014 (PMC10315752; doi:10.1002/cam4.6014)

Supplementary Figure 1

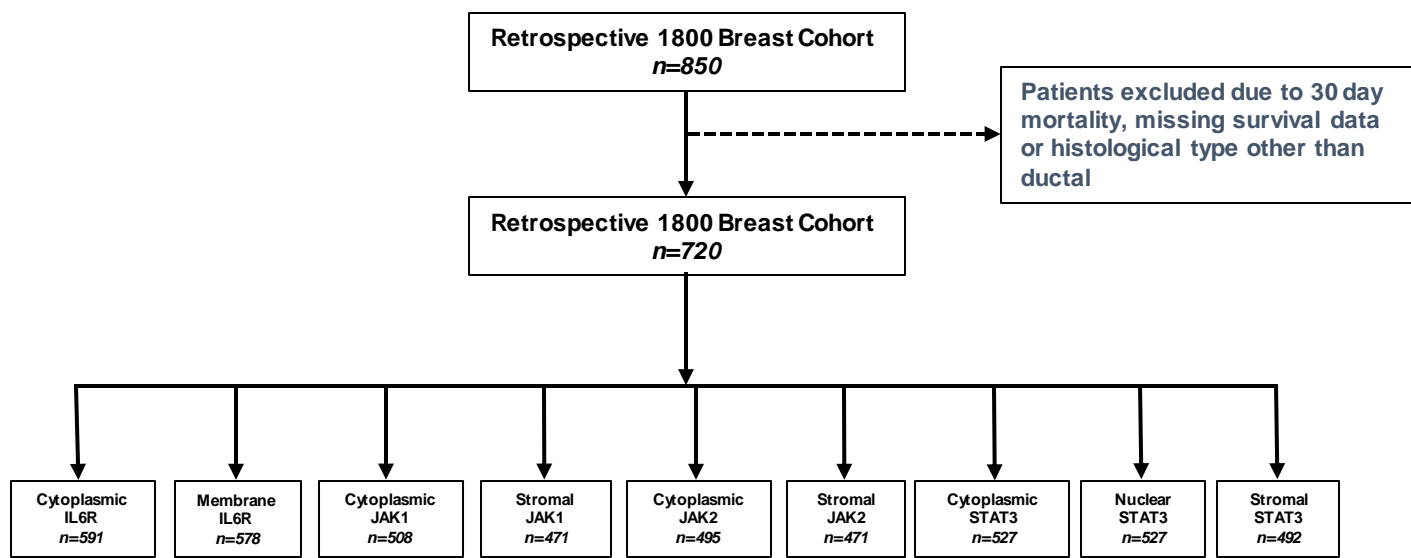

Supplement: Supplementary file 1 — Figure S1: [file CAM4-12-13225-s004.pdf]

Supplementary Figure 2

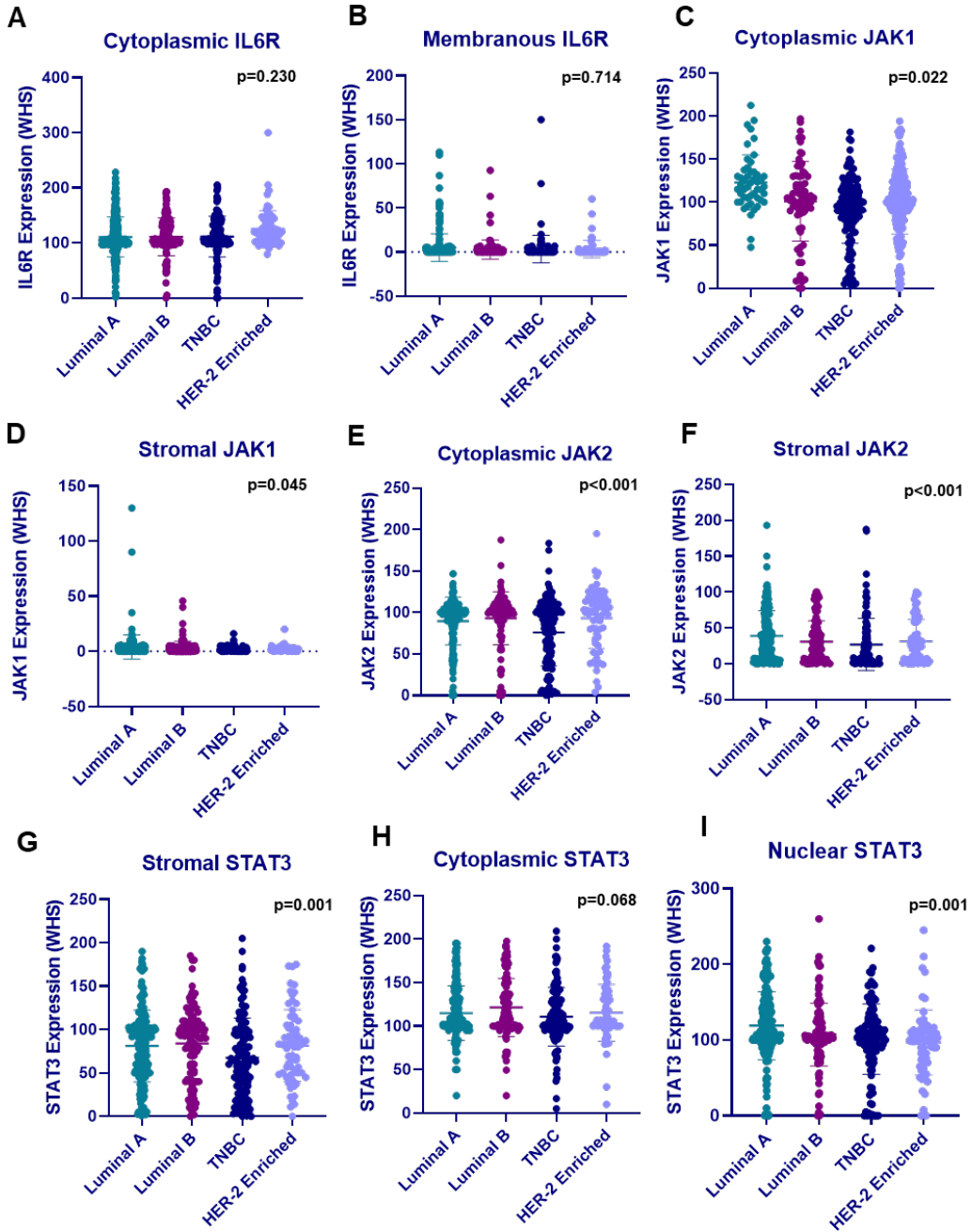

Supplement: Supplementary file 2 — Figure S2: [file CAM4-12-13225-s002.pdf]

Supplementary Figure 3

A

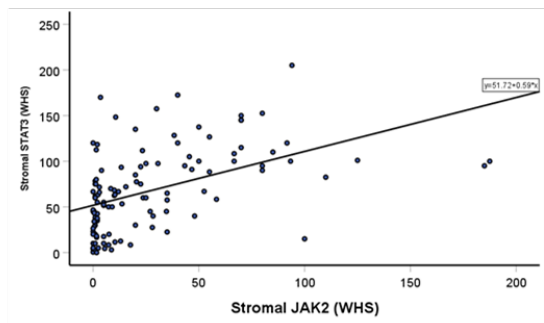

B

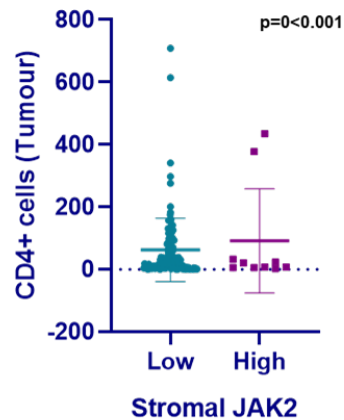

C

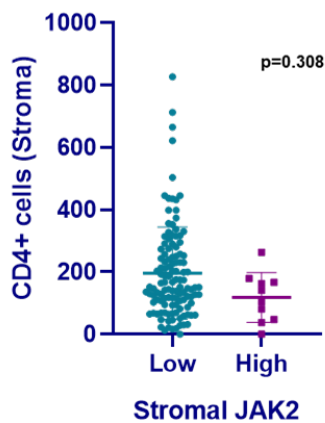

Supplement: Supplementary file 3 — Figure S3: [file CAM4-12-13225-s001.pdf]

Supplementary Figure 4

A

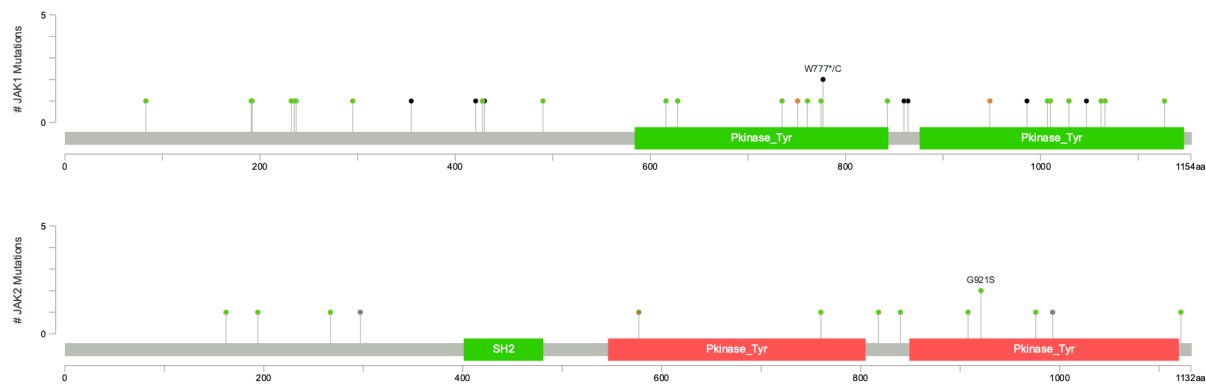

B

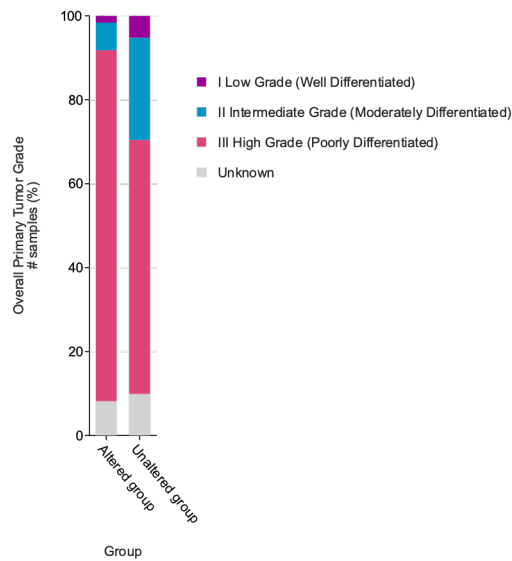

C

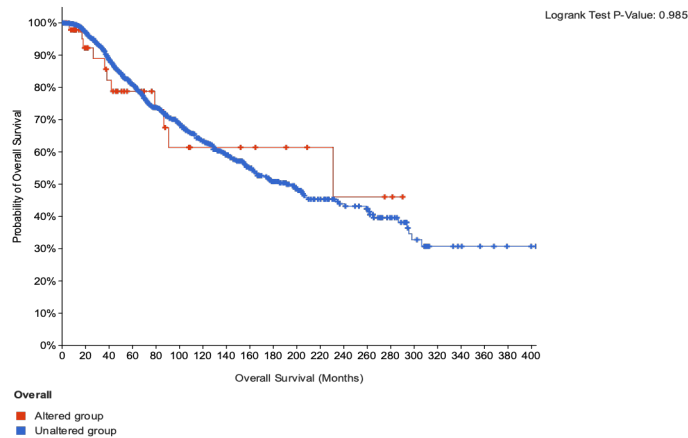

Supplement: Supplementary file 4 — Figure S4: [file CAM4-12-13225-s003.pdf]
